# Case report: Pulmonary hypertensive crisis leading to cardiac arrest during endoscopic evaluation in a 6-year-old boy with autism, severe malnutrition, and undiagnosed scurvy

**DOI:** 10.3389/fped.2022.1008507

**Published:** 2022-10-28

**Authors:** Laura A. Quinn, Stephanie P. Gilley, Allison D. Ta, Benjamin S. Frank, Carolyn B. Foley, Jaime M. Moore

**Affiliations:** ^1^ Department of Pediatrics, Section of Pediatric Gastroenterology, Hepatology and Nutrition, University of Colorado School of Medicine, Aurora, CO, United States; ^2^ Department of Pediatrics, Section of Nutrition, University of Colorado School of Medicine, Aurora, CO, United States; ^3^ Department of Pediatrics, Section of Cardiology, University of Colorado School of Medicine, Aurora, CO, United States; ^4^ Department of Anesthesiology, Division of Pediatric Anesthesiology, University of Colorado School of Medicine, Aurora, CO, United States

**Keywords:** scurvy, pulmonary hypertension (PAH), ARFID, autism, vitamin C deficiency, risk stratifcation, malnutrition, micronutrient deficiencies

## Abstract

Pediatric gastroenterologists are often responsible for the evaluation of malnutrition in the setting of selective eating. Endoscopic evaluation for conditions including eosinophilic esophagitis and celiac disease can help to identify and treat mucosal disease contributing to food selectivity. However, undiagnosed micronutrient deficiencies can cause cardiovascular derangements that significantly increase a patient's anesthetic risk. Vitamin C deficiency in particular, alone or in combination with severe malnutrition, is associated with a severe but reversible form of pulmonary arterial hypertension that, while life threatening in the acute phase, may significantly improve within days of starting ascorbic acid replacement therapy. Here we present a case of a 6-year-old boy with autism spectrum disorder (ASD), severe malnutrition, and undiagnosed chronic vitamin C deficiency who developed a pulmonary hypertensive crisis after induction of general anesthesia leading to cardiac arrest during endoscopic evaluation. While the association between food selectivity among youth with neurodevelopmental differences and vitamin C deficiency is well-described, and pulmonary hypertension is a recognized rare complication of scurvy, extant literature has not addressed next steps to improve patient outcomes. Using this case report as a foundation, we discuss specific patient populations to screen and treat for micronutrient deficiencies prior to anesthesia and propose a novel clinical algorithm for pre-anesthesia risk stratification and mitigation in patients specifically at risk for scurvy and associated pulmonary hypertension.

## Case

A 6-year-old boy with developmental delay and concern for autism presented to gastroenterology clinic with poor weight gain in the setting of chronic restrictive eating. His diet had become increasingly restrictive after choking on a french fry at two years of age, and consisted of a nutritionally complete supplemental beverage and candy. In the six months prior to presentation, he further narrowed his diet to only chocolate peanut butter cups and water. He had no net weight gain in the 21 months prior to presentation, and a ∼5% weight loss in the preceding three months. Body mass index (BMI) *z*-score had accordingly fallen from a max of −0.16 at four years of age to −6 at presentation. He presented to a local emergency department for inability to bear weight on the left leg one week prior to presentation with plain x-rays showing diffuse demineralization without fracture. Parents brought him to clinic in a stroller. Other past medical history included congenital nystagmus. The family history was only significant for asthma. There had been sporadic contact between the patient and healthcare system in the setting of vulnerabilities with housing, transportation, and the COVID-19 pandemic. Physical exam revealed cachexia. The patient had minimal verbal communication and the exam was further limited by combative and uncooperative behavior.

Both avoidant restrictive food intake disorder (ARFID) and eosinophilic esophagitis (EoE) were considered as possible root causes of his restricted diet. There was concern for refeeding syndrome, but also worry that he would not tolerate outpatient blood draws or nasogastric (NG) tube placement given his tactile defensiveness. As a result, the plan was made to bundle drawing labs, performing an upper endoscopy, and placing a bridled NG tube all under anesthesia followed by an admission for nutrition rehabilitation and monitoring.

**Figure 1:**
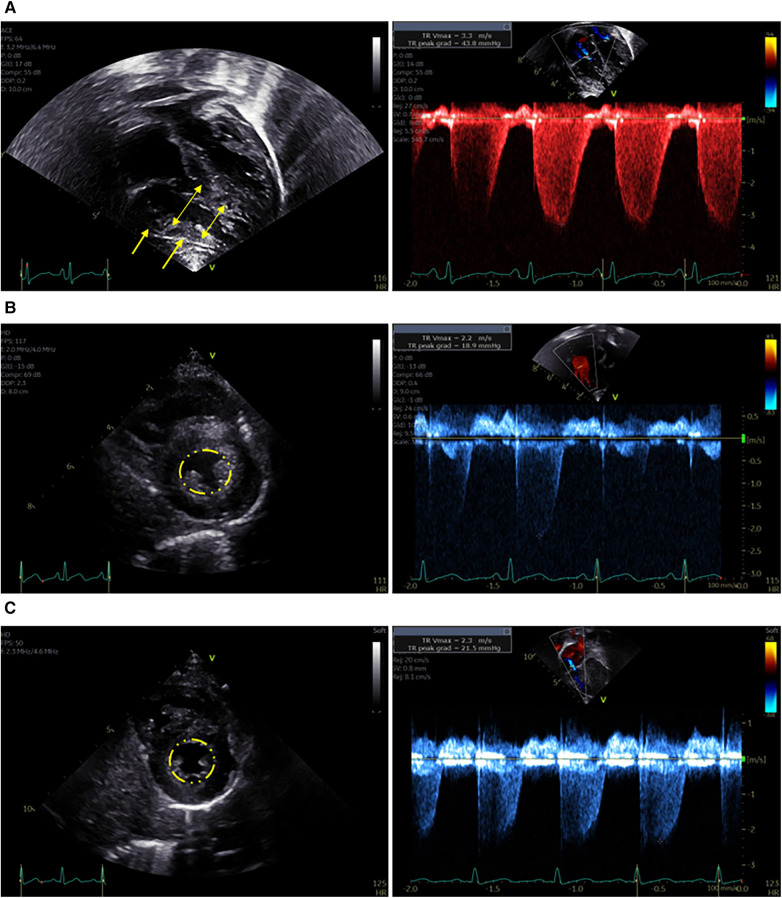
Representative images from echocardiograms obtained during the patient's course. (**A**) Immediately after cardiac arrest while on ECMO. Left panel: Apical 4-chamber view demonstrates right ventricular dilation (double headed arrows) and hypertrophy (single headed arrows). Right panel: Increased peak velocity of the tricuspid valve regurgitation jet indicates elevated right ventricular pressure. (**B**) Four weeks after cardiac arrest – on sildenafil. Left Panel: Parasternal short axis image demonstrates normal end-systolic septal position (indicated by circle), normal right ventricular size, and mild right ventricular hypertrophy. Right Panel: Normal peak velocity of the tricuspid valve regurgitation jet indicates normal right ventricular pressure. (**C**) Nine months after cardiac arrest – off sildenafil. Left Panel: Parasternal short axis image demonstrates normal end-systolic septal position (indicated by circle), normal right ventricular size, and no right ventricular hypertrophy. Right Panel: Normal peak velocity of the tricuspid valve regurgitation jet indicates normal right ventricular pressure.

The upper endoscopy was grossly unremarkable, but was quickly aborted after the patient developed bradycardia, hypotension, and then cardiac arrest. Labs immediately prior to the arrest showed no significant electrolyte abnormalities (potassium 4.8 mmol/L, normal 3.4–4.7 mmol/L), though bicarbonate (15 mmol/L, normal 20–31 mmol/L) and albumin (3.4 g/L, normal 3.5–5.2 g/L) were low. Despite resuscitation with intermittent return of spontaneous circulation, he suffered multiple pulseless electrical activity (PEA) arrests and was cannulated onto venoarterial extracorporeal membrane oxygenation (VA-ECMO). Echocardiogram shortly post-arrest showed signs of pulmonary arterial hypertension (PAH) including not only elevated tricuspid regurgitation peak velocity and severely diminished right ventricular function, but also significant right ventricular hypertrophy, suggesting that PAH predated the cardiac arrest (Figure 1A).

Given suspicion for a micronutrient deficiency driving his PAH, he was started empirically on vitamin C, thiamine, and a multivitamin within 24 h of admission to the pediatric intensive care unit (PICU). Selenium was started on day two. Nutrition assessment was consistent with severe malnutrition and multiple micronutrient deficiencies including vitamin A (15 mcg/dl, normal 19–77 mcg/dl), vitamin C (<0.1 mg/dl, normal 0.4–2 mg/dl), vitamin D (<3.5 ng/ml, normal 30–96 ng/ml), iron deficiency without anemia (ferritin 16.6 ng/ml, normal ≥20 ng/ml; iron saturation 12%, normal 20%–55%), selenium (43 ng/ml, normal 70–150 ng/ml), thiamine (55 nmol/L, normal 70–180 nmol/L), and zinc (0.39 mcg/ml, normal 0.6–1.2 mcg/ml). Other diagnoses on the differential for the cardiac arrest that were ultimately excluded included tachyarrhythmia (no evidence on cardiac monitor during the event), cardiac tamponade (no evidence on echocardiogram or clinically), pulmonary embolism (normal CT scan), and underlying primary neurologic or genetic/metabolic disorders. Given this, scurvy-associated PAH was thought to be the primary contributor to his arrest.

He was successfully decannulated from VA-ECMO on hospital day four, and was transitioned from inhaled nitric oxide to sildenafil on hospital day nine. At that point serum vitamin C had normalized after repletion (Table 1), and a repeat echocardiogram showed interval improvement in right ventricular dilation and tricuspid regurgitation. On hospital day 13, he underwent uncomplicated general anesthesia for gastrostomy tube (G-tube) placement and extensive dental work. He was found to have thalamic and cerebral hemorrhagic and ischemic strokes after the cardiac arrest and ECMO and spent a total of six weeks in the hospital including rehabilitation before being discharged on G-tube feeds, sildenafil, a multivitamin, zinc, and ferrous sulfate. A repeat echocardiogram prior to discharge showed only persistent right ventricular hypertrophy (Figure 1B), and repeat selenium and thiamine levels were normal.

**Table 1:** Interval change in select micronutrients after supplementation.

	Labs obtained hospital day 1:	Labs obtained hospital day 9–14:	Normal ranges:
Ascorbic acid (mg/dl)	<0.1	1.2	0.4–2
Selenium (ng/ml)	43	131	70–150
Thiamine (nmol/L)	55	141	70–180
Vitamin D (ng/ml)	<3.5	27.6	30–96

**Figure 2:**
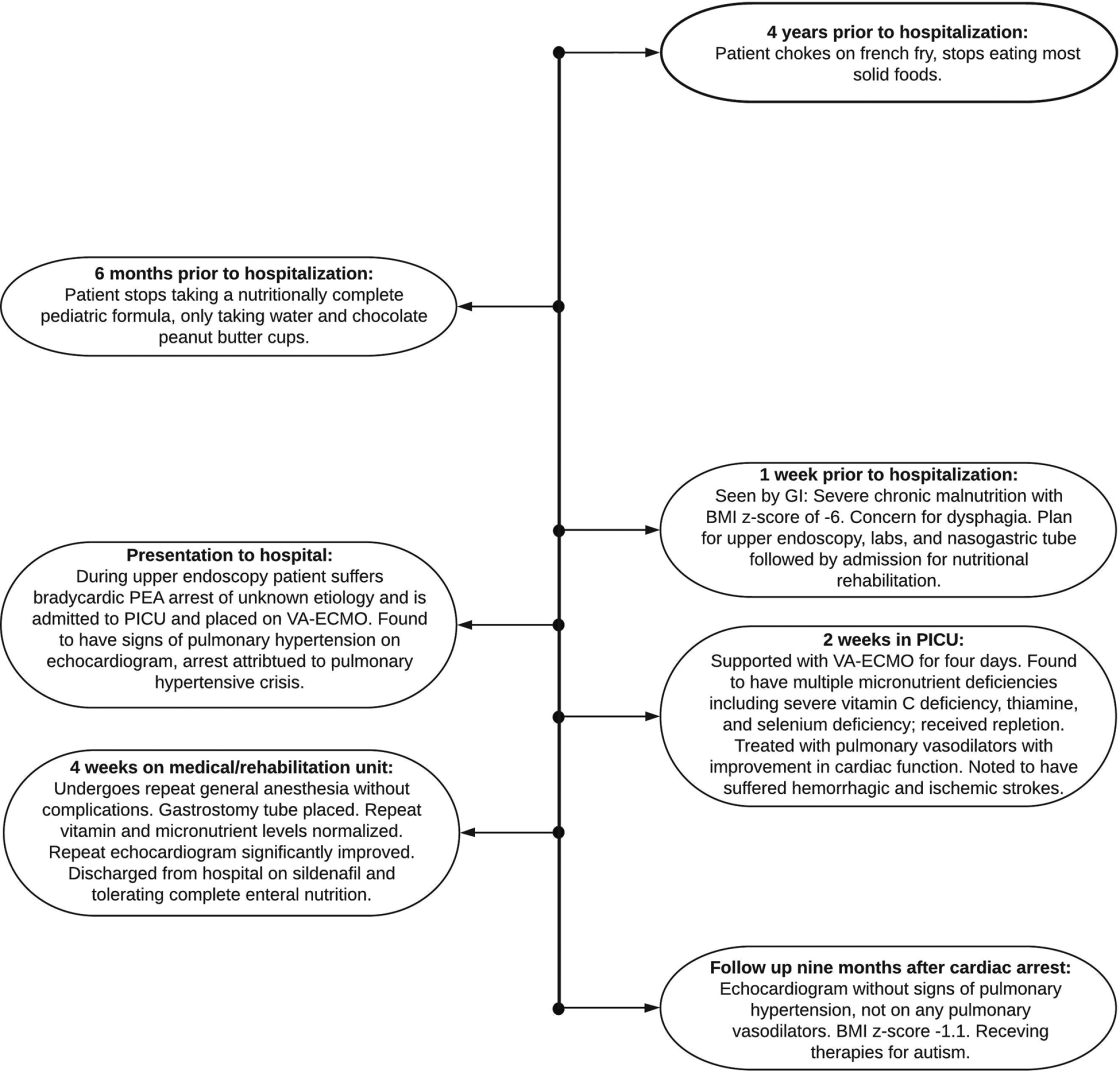
Timeline of case.

At follow-up three months later, he was receiving the majority of his nutrition *via* G-tube and his BMI *z*-score had improved to −0.8. He had no signs or symptoms of cardiac dysfunction including pallor, cyanosis, chest pain, dyspnea, peripheral edema, or syncope, and showed no evidence of PAH on repeat echocardiogram. Sildenafil was discontinued six months after the cardiac arrest and a follow up echocardiogram three months off sildenafil remained stable (Figure 1C). He continues to have increased muscle tone on the left side but without functional limitations. He is progressing with both physical and feeding therapy and is receiving support for autism which was formally diagnosed during his hospitalization. A timeline of the case is outlined in Figure 2.

## Discussion

### Common nutrition deficiencies in children with autism

The majority of children with autism spectrum disorder (ASD) have feeding difficulties, which occur five times more often than in children with other neurodevelopmental disorders and 15 times more often than in typically developing children (1). The most common of these are selective intake and ritualistic eating behaviors which are thought to be driven by sensory sensitivities to food texture, color, or taste, as well as fear of new foods and insistence on sameness (2–4). Additionally, children with ASD exhibit higher degrees of selectivity that persist longer than in neurotypical children (5, 6), which puts them at increased risk for growth faltering, nutrition deficiencies, dependence on supplemental nutrition, and impairments in psychosocial functioning (7). Patients who develop these downstream sequelae may meet the criteria for a concomitant diagnosis of avoidant restrictive food intake disorder (ARFID), which is increasingly recognized in combination with ASD (2, 8). Importantly, EoE is also more prevalent among children with ASD, and may contribute independently to food selectivity and possibly nutritional deficiencies (9, 10). Symptoms that help distinguish EoE from ARFID alone, such as dysphagia or abdominal pain in EoE and feeding indifference in ARFID, can be difficult to elicit in children with ASD, requiring a high level of clinical suspicion (11).

The co-occurrence of ASD and severe food restriction is associated with a number of nutrition complications (12). Depending on the caloric value and quantity of preferred foods consumed, highly restrictive eating can manifest with either unhealthy weight loss or weight gain (13). Regardless of body weight, children consuming a limited repertoire of foods are at risk for micronutrient deficiencies including vitamin A, vitamin B12, vitamin C, vitamin D, and thiamine (12, 14). In one systematic review, vitamin C deficiency was the most common vitamin deficiency among children with ASD and ARFID and presented earliest, manifesting as soon as one month after low (<10 mg/day) intake of vitamin C (12). Furthermore, even children using supplemental nutrition drinks or pediatric formula can be at risk for micronutrient deficiencies, particularly if the minimum volume needed to meet growth targets is lower than the intake required to meet micronutrient needs (15). Separate micronutrient supplementation is often warranted in these cases.

### Manifestations of vitamin C deficiency

Scurvy, the clinical syndrome resulting from vitamin C (ascorbic acid) deficiency, has been called “the great mimicker” because of its variable presentations. Vague constitutional symptoms like loss of appetite, fatigue, and irritability are common early manifestations and are followed by the development of cutaneous, hematologic, and musculoskeletal manifestations (16, 17). While scurvy is a well-described condition, it is difficult to recognize and diagnosis is often delayed (18, 19). Vitamin C is a water-soluble vitamin that humans cannot synthesize or store long term in the body. Plasma concentrations of ascorbic acid track closely with dietary intake of vitamin C-rich foods, like fruits and vegetables. Clinical manifestations of vitamin C deficiency develop after serum ascorbic acid levels fall below 0.2 mg/dl and total body value falls below 300 mg (20).

There are a number of characteristic clinical signs that should increase the index of suspicion for scurvy. Musculoskeletal manifestations affect the vast majority (80%–100%) of patients with recognized scurvy and particularly affect the lower extremities (20). Patients can present with refusal to bear weight or “pseudoparalysis” in the setting of subperiosteal bleeding or hemarthrosis (20, 21). Bone demineralization is the most common radiographic sign. Cutaneous manifestations include corkscrew hairs and follicular hyperkeratosis, as well as petechiae, ecchymoses, and perifollicular hemorrhage (22, 23). Gingival bleeding and hyperplasia are characteristic signs. Anemia affects 50% of patients with scurvy, and is often accompanied by concurrent iron and folate deficiencies (20). Rarely, unrecognized and untreated scurvy can be life-threatening in the setting of bleeding complications (cerebral hemorrhage and hemopericardium) and cardiopulmonary complications (24, 25).

Cardiopulmonary complications described in the setting of vitamin C deficiency range from exertional fatigue to cardiopulmonary collapse during anesthesia in the setting of right-sided heart failure due to PAH (17, 26–30). In previous reports of vitamin C deficiency and cardiopulmonary instability during anesthesia, commonalities among cases include a highly restrictive diet, comorbid ASD, and the presence of other clinical signs suggestive of scurvy (16, 17, 26).

### Pathophysiology of cardiopulmonary complications in vitamin C deficiency

Vitamin C deficiency is thought to contribute to the development of PAH through multiple mechanisms including impairments in nitric oxide production, oxygen sensing, and turnover of reactive oxygen species. PAH arises in the setting of vitamin C deficiency in part due to insufficient endogenous endothelial nitric oxide generation that leads to increased vascular tone in the pulmonary arteries (16, 17, 26, 31). Nitric oxide is generated by the conversion of L-arginine to L-citrulline by nitric oxide synthase, a process dependent on vitamin C (29). There is also evidence suggesting the hypoxia-inducible family of transcription factors may be inappropriately activated in patients with vitamin C deficiency, leading to a “pseudohypoxic state” that promotes the development of PAH (17, 26, 32, 33). Additionally, vitamin C plays a role in scavenging reactive oxygen species, whose presence compounds PAH (29, 34).

Furthermore, impairments in the production of catecholamines and cortisol in vitamin C deficiency blunt protective compensatory responses. Vitamin C is an important cofactor for dopamine β-hydroxylase and peptidylglycine α-amidating monooxygenase, which are key enzymes in the production of dopamine, epinephrine, norepinephrine, and vasopressin (35, 36). Finally, ascorbic acid is implicated in cortisol production. It is secreted locally by the adrenal glands in response to ACTH and precedes cortisol release (37, 38).

### Contribution of multiple nutrition risk factors to cardiopulmonary complications

In addition to vitamin C deficiency, other components of a patient's nutrition status may also contribute to the risk of cardiovascular complications during anesthesia and procedures. Published data are lacking on specific interactions among global nutrition status, multiple micronutrient deficiencies, and cardiopulmonary risk. However, having more than one nutrition risk plausibly increases the potential for adverse events and warrants additional assessment, treatment, and monitoring.

Severe acute or chronic malnutrition impacts nearly every organ system. Malnutrition impairs a patient's ability to tolerate and recover from anesthesia and invasive procedures for several reasons. Malnutrition increases risk of infection, diminishes wound healing and skin integrity, blunts the cortisol stress response, and decreases intestinal absorption (39). Several studies have also documented impaired left ventricular function that correlates with the degree of malnutrition (40, 41).

Administration of dextrose-containing intravenous fluids in patients with severe malnutrition can precipitate the refeeding syndrome, characterized by hypophosphatemia, hypokalemia, and/or hypomagnesemia that can result in impaired cardiac and respiratory function. Refeeding syndrome can further induce retention of sodium and fluids, leading to pulmonary edema and cardiac overload (39, 42).

Thiamine deficiency, called beriberi, manifests primarily with neurologic and psychiatric symptoms including impaired memory, confusion, apathy, irritability, decreased deep tendon reflexes and peripheral neuropathy (43). It may also present with congestive heart failure and dilated cardiomyopathy (wet beriberi) and may rarely be associated with PAH (44, 45). In addition, there have been reports of severe thiamine deficiency presenting as cardiac arrest in pediatric patients with autism (29, 46).

Selenium deficiency is known to negatively impact cardiovascular health, including increased risk for cardiomyopathy, heart failure, and atherosclerosis (47). Associations between selenium and cardiovascular function have been reported in unsupplemented patients after bariatric surgery (48), as well as in Keshan disease, an endemic cardiomyopathy in rural China (49). Possible mechanisms of selenium in cardiovascular function were recently reviewed (47). Selenium is found in the soil in quantities that vary geographically and impact a population's selenium levels. Soil selenium levels are expected to decrease as global temperatures increase, which may exacerbate deficiency in high risk populations (50). There is additional evidence that pyridoxine (vitamin B6) and vitamin D deficiencies may also play a role in development of cardiovascular disease (43).

Multiple micronutrients play a role in the development of anemia: in particular iron, but also folate, vitamin B12, copper (51), and vitamin E (52). Mild or moderate chronic anemia may present with fatigue, pallor, exercise intolerance, tachycardia, and/or syncope, or may be asymptomatic due to compensatory mechanisms. Severe chronic anemia can cause growth failure and neurodevelopmental impairment. Patients with severe anemia are at increased risk of heart failure (51) and adverse outcomes related to pre existing heart conditions.

The patient case presented here involved not only vitamin C deficiency, but also deficiencies in vitamin D, selenium, and thiamine. The cumulative impact of multiple micronutrient deficiencies that can adversely affect cardiac function, superimposed on severe malnutrition, may have contributed to his severe presentation.

### Pre-Anesthesia risk stratification and mitigation

**Figure 3:**
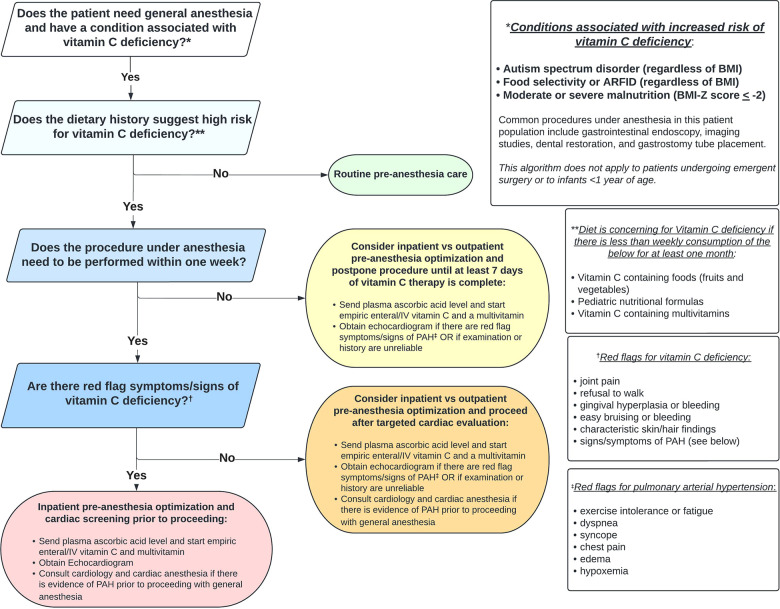
Approach to pre-anesthesia evaluation in patients at risk for vitamin C deficiency.

Using a systematic interdisciplinary approach to evaluate patients at risk for vitamin C deficiency who require anesthesia can facilitate risk stratification, screening, and medical optimization, which has the potential to reduce adverse events. Figure 3 outlines one such approach using a novel clinical algorithm. Implementation requires coordination among gastroenterology/nutrition, anesthesia, and cardiology providers and could also include clinicians who request or carry out procedures under anesthesia (e.g. primary care, radiology, dentistry, surgery).

The first step is recognizing if the patient is at increased risk for vitamin C deficiency. Any condition that results in less than weekly consumption of vitamin C from foods, fortified formulas, or vitamins for one month or longer should raise suspicion of vitamin C deficiency. Diagnoses where this presentation is common include ASD and ARFID (across the BMI spectrum), and acute or chronic moderate to severe malnutrition. In addition to endoscopy, other procedures that may require general anesthesia in these patient groups include imaging (e.g., MRI), dental restoration, and G-tube placement. If the patient does not have increased risk for vitamin C deficiency, routine pre-anesthesia care can be followed.

If the patient is at high risk for vitamin C deficiency, determining the urgency for completing the procedure should next be made. Significant improvement in the hematologic, musculoskeletal, and cardiopulmonary manifestations of scurvy has been documented within one week of vitamin C supplementation (16). Thus, if the procedure can be postponed for seven days or longer, we recommend medical optimization (inpatient or outpatient), including seven days of vitamin C repletion, prior to exposure to general anesthesia. Factors that may favor admitting the patient to the hospital include inability/unwillingness of the child to take oral vitamin supplements, the need for NG tube placement, intravenous (IV) access for hydration, vitamin supplementation, and/or lab draws, and monitoring for refeeding syndrome. Regardless of the setting for medical optimization, we recommend obtaining a plasma ascorbic acid level and starting empiric vitamin C and multivitamin supplementation *before* the level comes back (or if the level cannot be obtained). Recommended pediatric vitamin C dosing for scurvy is 300 mg for one week to achieve adequate plasma concentrations, followed by 100 mg for one to three months for tissue saturation (53, 54). Additionally, if the patient has red flag symptoms or signs consistent with PAH and/or if the physical exam or history are unreliable (e.g., based on neurodevelopmental limitations), we recommend obtaining a screening echocardiogram.

If the procedure under anesthesia must be performed within seven days, it is important to determine if the patient has red flag symptoms or signs of vitamin C deficiency: joint pain/change in ambulation, bleeding/bruising, or pathognomonic skin or hair findings. If there are none, we recommend obtaining a screening echocardiogram only in those patients with red flag symptoms/signs of PAH or if the physical exam or history are unreliable. If there are signs of PAH on echocardiogram, cardiology should be consulted prior to proceeding. Given low risk and potential high yield, we still recommend obtaining a plasma ascorbic acid level and starting empiric vitamin C and multivitamin supplementation as early as possible before proceeding with anesthesia in this group of patients.

Finally, if the procedure under anesthesia must be performed within seven days and the patient has red flag symptoms or signs of vitamin C deficiency, we recommend inpatient admission to obtain the plasma ascorbic acid level, to start empiric vitamin C (IV route may be preferable to bypass the enteral absorption ceiling) and multivitamin supplementation, and to perform a screening echocardiogram (55). Again, if the echocardiogram shows evidence of PAH, cardiology should be consulted and a plan with cardiac anesthesia devised before proceeding. PAH may be responsive to inhaled nitric oxide therapy while vitamin C is being repleted (17).

Anesthetic goals in this scenario include avoidance of hypoxia, hypercarbia, acidosis as well as a balanced induction of general anesthesia to avoid hypotension and low cardiac output. If a patient with known PAH shows evidence of pulmonary hypertensive crisis while under general anesthesia, vasopressors should be selected carefully with the goals of decreasing pulmonary vascular resistance, supporting cardiac inotropy, and avoiding systemic hypotension. Multiple options are appropriate depending on the clinical circumstance.

## Conclusion

Patients with highly selective feeding are at risk for micronutrient deficiencies, independent of weight status. Multiple micronutrient deficiencies, but in particular vitamin C, predispose to adverse events during anesthesia including pulmonary hypertensive crisis, hypotension, and cardiac arrest. Our novel clinical algorithm provides guidance for evaluation of patients with selective eating to identify pre-existing cardiopulmonary impairment prior to anesthesia.

## Data Availability

The original contributions presented in the study are included in the article/Supplementary Material, further inquiries can be directed to the corresponding author/s.
